# Rare but serious: pulmonary vascular disease around the world

**DOI:** 10.1016/j.jhlto.2025.100327

**Published:** 2025-07-18

**Authors:** Clara Hjalmarsson, Arun Jose, Hooman Poor, Camila M.C. Loureiro, Valentina Stosor, Tomas Pulido, Mrinalini Krishnan

**Affiliations:** a Department of Cardiology, Sahlgrenska University Hospital, Gothenburg, Sweden; b Institute of Medicine at Sahlgrenska Academy, University of Gothenburg, Gothenburg, Sweden; c Division of Pulmonary, Critical Care, and Sleep Medicine, University of Cincinnati, Cincinnati, OH; d Division of Pulmonary, Critical Care and Sleep Medicine, Icahn School of Medicine at Mount Sinai, New York, NY; e Pulmonary Medicine, Santa Casa da Bahia, Salvador, BA, Brazil; f Department of Medicine, Federal University of Bahia, Salvador, BA, Brazil; g Northwestern University Feinberg School of Medicine, Chicago, IL; h Ignacio Chavez National Heart Institute, Mexico City, Mexico; i MedStar Heart and Vascular Institute / Georgetown University School of Medicine, Washington, DC

**Keywords:** Portopulmonary hypertension, HIV-PAH, Schistosoma-associated PAH, High-altitude PH, Chronic hemolytic anemia associated PH

## Abstract

Pulmonary arterial hypertension (PAH), a subtype of pulmonary hypertension (PH) classified under Group 1 of the World Health Organization (WHO) classification, is a progressive disorder characterized by pulmonary vascular remodeling, increased pulmonary vascular resistance, and eventual right heart failure. While common etiologies are well described, less frequent causes are often underrecognized, despite their potential impact on prognosis and therapeutic decision-making. During the April 2025 International Society of Heart and Lung Transplantation (ISHLT) annual meeting in Boston, USA, a dedicated symposium entitled “Rare but Serious: Pulmonary Vascular Disease Around the World” addressed these overlooked forms of pulmonary vascular disease (PVD). This review summarizes the latest diagnostic and therapeutic insights into several rare but clinically significant entities, including portopulmonary hypertension (PoPH), PH associated with hematologic disorders, HIV-associated PAH, high-altitude PH and PAH, and schistosomiasis-associated PAH (Sch-PAH). Raising awareness and understanding of these conditions is critical to ensuring timely diagnosis, personalized treatment, and improved patient outcomes.

Unusual causes of pulmonary arterial hypertension (PAH) or pulmonary hypertension (PH) are frequently underrecognized in clinical practice, often due to their rarity, overlapping clinical features, and limited awareness among healthcare providers.^1^ However, correctly identifying these etiologies is critical, as they may carry distinct pathophysiological mechanisms, prognostic implications, and therapeutic opportunities.

At the latest annual meeting of the **International Society of Heart and Lung Transplantation** held in Boston, USA, in April 2025, a dedicated Pulmonary Vascular Disease symposium, **“Rare but Serious: Pulmonary Vascular Disease Around the World”**, addressed some of these less common types of pulmonary vascular disease (PVD), including portopulmonary hypertension (PoPH), PH from hematologic diseases, HIV-associated PAH (HIV-PAH), high altitude PH (HAPH) and PAH (HA-PAH), and schistosomiasis-associated PAH (Sch-PAH). Increased awareness and timely recognition of these rare forms of PH not only enable more accurate diagnosis but also allow for individualized treatment strategies that can improve outcomes and quality of life for affected patients. This article highlights the latest diagnostic and therapeutic approaches of these conditions (Table 1).

**Table 1:** Clinical Characteristics, Lab findings and Treatment, by Type of Pulmonary Hypertension (PH)

Type of PH	Clinical Features	Diagnostic Tools (in Addition to Echocardiography and Right Heart Catheterization)	Treatment
Portopulmonary Hypertension (PoPH)	Signs of portal hypertension (esophageal varices, caput medusae, ascites, and splenomegaly) Right heart failure (late stage)	Liver function tests Abdominal imaging – liver ultrasound with doppler, CT-abdomen Right heart catheterization with hepatic venous gradient measurement	PAH-targeted therapy Liver transplantation (select cases)
PH in Hematologic Disorders	Hemolysis-related symptoms (jaundice, dark urine, elevated lactate dehydrogenase (LDH) indicating ongoing red cell destruction); Hepatosplenomegaly Vaso-occlusive crisis in sickle cell disease	Complete blood counts (CBC) with peripheral smear, lactate dehydrogenase (LDH), reticulocyte count, bilirubin, haptoglobin	Treat underlying hematologic disorder PAH-targeted therapy Blood transfusions Oxygen therapy
Schistosomiasis-Associated PAH	Hemoptysis (may occur due to rupture of bronchial-pulmonary collaterals or elevated pulmonary pressures) Signs of portal hypertension (esophageal varices, caput medusae, hepatosplenomegaly, and thrombocytopenia secondary to splenic sequestration) Clubbing and cyanosis (late stage)	Schistosoma antibody serology Abdominal imaging – liver ultrasound with doppler, CT-abdomen	PAH-targeted therapy Antiparasitic treatment -praziquantel Supportive care
HIV-Associated PAH	Ascites and hepatomegaly (in right- heart failure and congestion) Lymphadenopathy	HIV viral load and CD4 count	PAH-targeted therapy Antiretroviral therapy Supportive care
High-Altitude PH (HAPH) and PAH (HA-PAH)	Polycythemia Dyspnea on exertion Cyanosis Fatigue	Hemoglobin/hematocrit, oxygen saturation	Descent to lower altitude Oxygen therapy Therapeutic phlebotomy PAH-targeted therapy in HA-PAH

## Portopulmonary hypertension and hepatopulmonary syndrome

This section covered the epidemiology, pathogenesis, and clinical features of two liver disease-associated pulmonary vascular disorders: **portopulmonary hypertension** (**PoPH**) and **hepatopulmonary syndrome** (**HPS**).

PoPH is a type of PAH that occurs exclusively in the context of portal hypertensive liver disease.^2^, ^3^ It is believed to affect approximately 5–6% of all patients with portal hypertension, and has exceptionally poor survival, with less than 40% of patients alive at 5 years, even with targeted PAH therapy. In contrast, HPS is characterized by arterial deoxygenation, hypoxemia, and intrapulmonary vascular dilatation with shunt physiology, with a comparable prevalence in patients with underlying liver cirrhosis, and approximately 20% survival at 5 years in the absence of liver transplantation.^4^, ^5^

Both conditions typically present in the 5th to 6th decades of life, with patients commonly reporting dyspnea, weakness, and fatigue. Patients with HPS may exhibit dyspnea (platypnea) or hypoxemia (orthodeoxia) that worsens in the upright position and improves with recumbency; however, neither feature is common nor pathognomonic for the condition. Physical exam findings may reflect the differing aspects of PVD in these conditions; patients with PoPH typically develop signs and symptoms of right heart failure in advanced stages, whereas those with HPS exhibit complications of chronic hypoxemia, such as digital clubbing and cyanosis (Table 2).^6^ Unfortunately, the molecular mechanisms driving disease pathogenesis in these conditions remain largely unknown.^7^

**Table 2:** Comparison of Clinical Characteristics and Criteria for Transplantation in HPS and PoPH. *Adapted from Jose A, McCormack F, Shah S, et al. Hepatopulmonary Syndromes in Orphan Lung Diseases: A clinical guide to rare lung disease. 2^*nd*^*Edition. London:Springer-Verlag*.

	**HPS**	**PoPH**
Clinical Features	Dyspnea, orthodeoxia (rare), platypnea (rare), weakness, fatigue	Dyspnea, weakness, fatigue, syncope, orthopnea
Physical Exam Findings	Digital clubbing, cyanosis Underlying sequelae of liver cirrhosis (peripheral edema, ascites, pulsatile liver)	Tricuspid regurgitant murmur, split second heart sound Underlying sequelae of portal hypertensive liver disease (peripheral edema, ascites, pulsatile liver)
Age at Diagnosis	40−50 years	60 years
Molecular Pathogenesis	Unknown, various factors (Endothelin 1, pulmonary nitric oxide, deficiency of bone morphogenetic protein type 9 and type 10, intestinal bacterial translocation into systemic circulation, genetic polymorphisms) have been implicated	Unknown, various factors (Endothelin 1, pulmonary nitric oxide, deficiency of bone morphogenetic protein type 9, excess circulating endoglin, estrogen metabolites, genetic polymorphisms) have been implicated
Portal Hypertension	Usually present	Always present
United States MELD Exception Criteria for Liver Transplantation	• Underlying chronic liver disease • Diagnostic arterial blood gas with PaO2 < 60 mmHg on room air • Intrapulmonary shunting (bubble-enhanced transthoracic echocardiography or macroaggregated albumin scan • Exclusion of pulmonary causes of hypoxemia	• Diagnosis of PoPH (mean pulmonary arterial pressure ≥ 25 mmHg, pulmonary vascular resistance ≥ 3 Wood Units) • Improvement with treatment: mean pulmonary arterial pressure 35−45 mmHg with pulmonary vascular resistance <3 Wood Units **OR** mean pulmonary arterial pressure < 35 mmHg with pulmonary vascular resistance < 5 Wood Units
Response to Liver Transplantation	Curative, indication for liver transplant	Beneficial, ∼50% resolution and weaning of targeted PAH therapy, not an indication alone for liver transplantation

Patients with HPS or PoPH may benefit from liver transplantation, and post-transplant outcomes are generally comparable to patients with liver cirrhosis without PVD. Liver transplantation offers a definitive cure and is therefore the preferred treatment for HPS.^8^, ^9^ Aside from liver transplantation, supplemental oxygen is the only known supportive therapy with benefit in HPS. In contrast, while liver transplantation can be beneficial in PoPH — with the majority improving after transplant, and up to 50% of patients able to discontinue targeted PAH therapy — a small subset of patients may deteriorate postoperatively. Transplantation is generally contraindicated in patients with a mean pulmonary arterial pressure (mPAP) ≥ 50, as this has historically been associated with significantly increased mortality.^10^, ^11^, ^12^ In lieu of liver transplantation, targeted PAH therapy remains the mainstay of treatment in PoPH, albeit with limited evidence from randomized controlled studies and small cohort studies.^13^, ^14^ As transplantation can be beneficial in both PoPH and HPS, specific criteria exist (which vary by geographic region) to help shorten waitlist times and expedite liver transplantation for patients with these conditions.^15^, ^16^

Despite the progress that has been made in the management of PVD due to liver disease, multiple gaps in knowledge persist, and patients afflicted with these conditions still suffer from unacceptably high morbidity and mortality. Future research is needed to clarify the molecular mechanisms by which liver disease can lead to distinct forms of PVD, improve prediction of transplant response in PoPH, reduce care disparities and enhance patient outcomes.

## PH associated with hematologic disorders

Hematologic disorders — particularly **chronic hemolytic anemia** and **myeloproliferative disorders** — are known to be a cause of PH. Until the 4th World Symposium on PH, these hematologic disorders were classified as WHO Group 1. However, because of the multifactorial and often unclear mechanisms of PH in these conditions, they have since been reclassified as WHO Group 5.^1^

**Chronic hemolytic anemia** include sickle cell disease (SCD), β-thalassemia, spherocytosis, stomatocytosis, autoimmune disorders, and paroxysmal nocturnal hemoglobinuria. All these entities can contribute to PH through various mechanisms, including increased cardiac output due to anemia (hyperdynamic circulation), diastolic heart failure leading to pulmonary venous congestion and post-capillary PH, and elevated pulmonary vascular resistance driven by diverse underlying processes. During hemolysis, hemoglobin and arginase are released from red blood cells, leading to decreased nitric oxide (NO), an intrinsic pulmonary vasodilator. Specifically, free hemoglobin inactivates NO and arginase depletes L-arginine, a substrate for NO synthesis.^17^ The prevalence of PH in SCD, the best-studied form of chronic hemolytic anemia causing PH, is 6–10%, with an associated increased mortality in SCD patients with PH.^18^ The Walk-PHaSST trial, a 16-week, double-blind, placebo-controlled trial of sildenafil in patients with SCD with PH and low exercise capacity was stopped early because of higher serious adverse events in the sildenafil group, specifically an increase in the rate of hospitalization for vasoocclusive crises.^19^

PH is also an uncommon and often underdiagnosed complication of **chronic myeloproliferative diseases**, such as polycythemia vera (PV), essential thrombocythemia (ET), primary myelofibrosis (MF), and chronic myeloid leukemia (CML).^20^ These may cause PH via three major mechanisms: pre-capillary PH, chronic thromboembolic pulmonary hypertension (CTEPH), and drug-induced PAH. Portal hypertension, intrathoracic extramedullary hematopoiesis, and increased circulating megakaryocytes, leading to microthrombosis from poor deformability and aggregation tendency may also contribute. Moreover, circulating megakaryocytes also release vasoactive products, which can result in pulmonary vascular remodeling.

Interestingly, patients with PV or ET are more likely to develop CTEPH, while patients with MF are more likely to have multifactorial PH (WHO Group 5 PH).^20^ In patients with MF and PH, successful allogeneic hematopoietic cell transplantation (alloHCT) seems to partially reverse the PH. However, PH is associated with inferior survival due to increased nonrelapse mortality in patients with MF undergoing alloHCT.^21^

Among drugs, Dasatinib, a tyrosine kinase inhibitor that inhibits BCR/ABL kinase, which is used for the treatment of CML, has been associated with the development of PAH. Dasatinib discontinuation improves symptoms and hemodynamics in PAH, though full resolution without PAH-specific therapy is rare.^22^

## Schistosomiasis-associated pulmonary arterial hypertension

**Figure 1:**
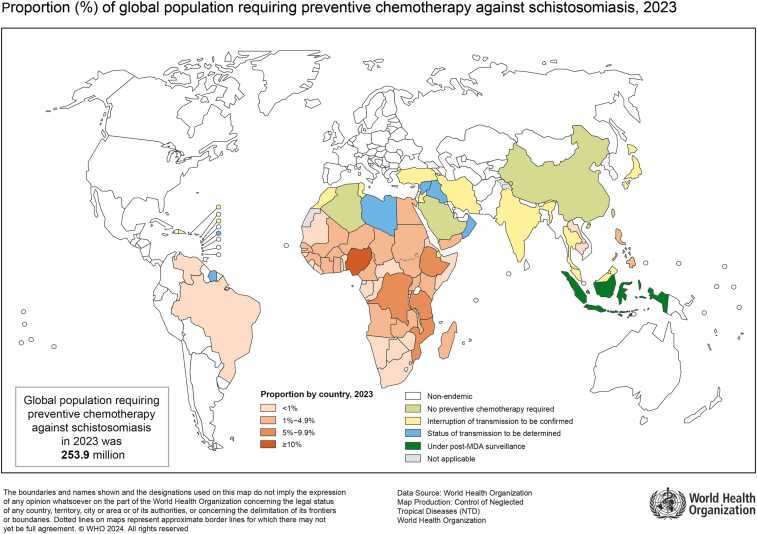
Proportion (%) of global population requiring preventive chemotherapy against schistosomiasis, 2023, according to WHO. Reproduced with permission by World Health Organization.

Schistosomiasis is a neglected tropical disease caused by the flatworm *Schistosoma* sp. Despite its clinical significance, schistosomiasis continues to be a public health problem, especially in the developing world. Most deaths and disease-related disabilities occur in Africa.^23^ Schistosomiasis is a parasitic infection that can progress from an asymptomatic form to serious clinical manifestations.^24^ Approximately 5–10% of patients with severe hepatosplenic schistosomiasis may develop **Schistosoma-associated pulmonary arterial hypertension** (Sch-PAH).^25^ In endemic areas, schistosomiasis causes PAH in about one million people worldwide (Figure 1). Sch-PAH is classified as group 1 PH, but it arises from a combination of several factors.^1^ Schistosoma egg embolization to the lung via portosystemic shunts results in mechanical obstruction, as well as a type-2 immune response, which drives vascular inflammation and remodeling along with increased shear stress on the pulmonary vasculature.^24^, ^26^

Screening for Sch-PAH should be guided by clinical signs of progressive right heart failure and echocardiographic indicators suggesting PH. A diagnosis of Sch-PAH requires a history of environmental exposure or schistosomiasis infection with or without prior treatment, accompanied by evidence of hepatosplenic involvement — most notably periportal fibrosis. Definitive diagnosis requires right heart catheterization confirming the presence of pre-capillary pulmonary hypertension.^25^

Sch-PAH occurs predominantly in females, typically diagnosed at 30 to 60 years of age. Clinical presentation for Sch-PAH patients is similar to idiopathic PAH,^27^, ^28^ however, pulmonary artery enlargement is more pronounced in Sch-PAH.^29^, ^30^ Patients with Sch-PAH notably have a more favorable hemodynamic profile and better survival than patients with idiopathic PAH.^26^ However, recent data from endemic areas in Brazil suggest that Sch-PAH is indistinguishable from other PAH etiologies, including by hemodynamic profile.^31^, ^32^ The effect of antiparasitic treatment in Sch-PAH is contentious, as a large number of patients with Sch-PAH do not carry the Schistosoma worm at the time of diagnosis.^25^ Experimental studies have shown that treatment with praziquantel can reverse PVD.^33^ Conversely, clinical studies have reported that PAH-targeted therapies are both safe and effective in patients with Sch-PAH, with improved survival outcomes.^34^, ^35^ Additional studies regarding Sch-PAH treatment, as well as epidemiology, hemodynamic presentation and prognosis, especially in endemic areas, are essential for a better understanding of this relevant disease.

## HIV-associated pulmonary arterial hypertension

**Figure 2:**
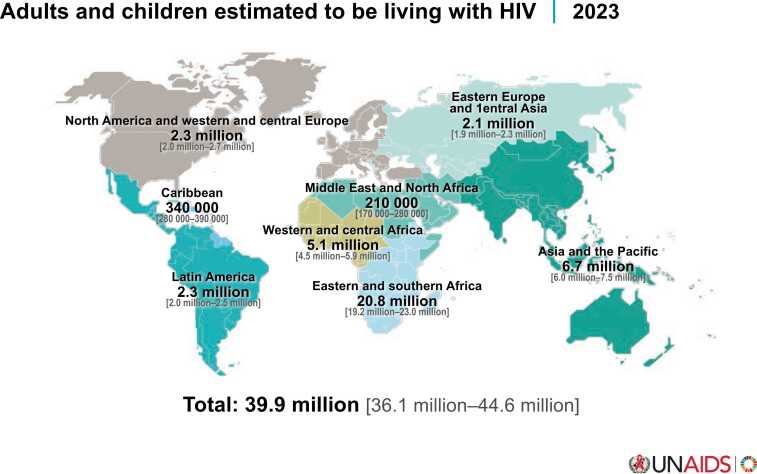
Global Burden of HIV Infection in 2023, according to UNAIDS. Reproduced with permission by The Joint United Nations Programme on HIV/AIDS.

There are 39.9 million people globally living with HIV, with the highest burden in Sub-Saharan Africa^36^ (Figure 2). With potent antiretroviral therapy, people with HIV now have near-normal life expectancy, but face earlier and higher rates of non-AIDS conditions, including coronary artery disease, heart failure, COPD, and PAH, compared to those without HIV.^37^, ^38^

**HIV-associated PAH** (HIV-PAH) is classified as WHO group 1.4.2.^1^ and has a reported prevalence of around 0.5%, but data are lacking from developing countries with the highest HIV rates.^39^ Recent results indicate a decreasing prevalence in the era of antiretroviral therapy (ART).^37^, ^40^ Though rare, HIV-PAH is 1000 times more common than idiopathic PAH and more frequently affects men and younger individuals.^37^ HIV-related factors associated with HIV-PAH include active viremia and CD4 lymphopenia.^40^, ^41^ Contributing or synergistic factors include co-pathogens such as hepatitis B and C viruses, human herpesvirus 8, cytomegalovirus, *Schistosoma*, and *Mycobacterium tuberculosis,* and substance and injection drug use (cocaine, methamphetamine, and opioids).^39^, ^42^

In HIV-PAH, lung histopathologic findings are similar to that of iPAH, including intimal and medial hyperplasia, concentric intimal fibrosis, and perivascular lymphocytic infiltrates. Classic plexiform lesions are present in up to 78% of cases.^39^ The pathogenesis of HIV-PAH is not due to direct infection of pulmonary artery endothelial cells (PAEC) or smooth muscle cells (PASMC), but rather the effect of viral proteins — Nef, gp120, Tat, and Vpr — which trigger pro-inflammatory and pro-oxidant states through mitochondrial dysfunction, lymphocyte apoptosis, cytokine release, and endothelin-1 secretion. These processes result in PAEC and PASMC proliferation.^37^, ^42^

Due to nonspecific early symptoms and high cardiopulmonary disease burden in patients with HIV, early echocardiography and further testing are essential for those with dyspnea, fatigue, or exercise intolerance, with prompt referral and treatment if PAH is diagnosed.

**Figure 3:**
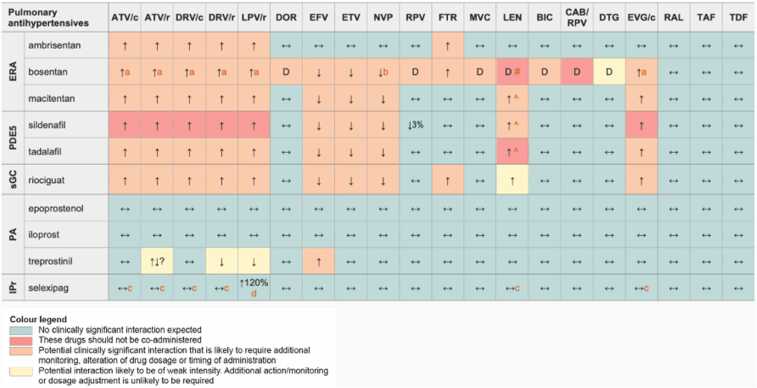
Drug Interactions Between Pulmonary Antihypertensives and Antiretroviral Agents. Reproduced with permission by the European AIDS Clinical Society. *Footnote tillFigure 3*. **Legend**: ↑ Potential elevated exposure of the pulmonary antihypertensive, ↓ Potential decreased exposure of the pulmonary antihypertensive, ↔ No significant effect D Potential decreased exposure of ARV drug E Potential elevated exposure of ARV drug. Numbers refer to increased or decreased AUC as observed in drug-drug interaction studies. **Pulmonary antihypertensive drug classes**: ERA, endothelin receptor antagonist; IPr, IP receptor agonists; PA, prostacyclin analogs; PDE5, phosphodiesterase type 5 inhibitors; sGC, soluble guanylate cyclase stimulators. **Antiretroviral agents**: ATV/c, atazanavir/cobicistat; ATV/r, atazanavir/ritonavir; DRV/c, darunavir/cobicistat; DRV/r, darunavir/ritonavir; LPV/r, lopinavir/ritonavir; DOR, doravirine; EFV, efavirenz; ETV, etravirine; NVP, nevirapine; RPV, rilpivirine; FTR, fostemsavir; MVC, maraviroc; LEN, lenacapavir; BIC, bictegravir; CAB/RPV, cabotegravir/rilpivirine long-acting injections; DTG, dolutegravir; EVG/c, elvitegravir/cobiscistat; RAL, raltegravir; TAF, tenofovir alafenamide; TDF, tenofovir disoproxil fumarate. No clinically relevant interactions expected with abacavir, emtricitabine, lamivudine, zidovudine, oral cabotegravir, and ibalizumab. **Comments. a** Co-administration is not recommended in the European labels, but the US labels suggest the following dose modifications: When starting bosentan in persons already on PI/b or EVG/c use a bosentan dose of 62.5 mg qd or every other day. Discontinue bosentan at least 36 h prior to starting PI/b or EVG/c and restart after at least 10 days at 62.5 mg qd or every other day. **b** Potential additive liver toxicity. **c** Exposure of parent drug increased but exposure of active metabolite unchanged. d This change is unlikely to be clinically relevant. ^ LEN causes moderate inhibition of CYP3A4 and, when discontinued, remains in the circulation for prolonged periods. Residual concentrations of LEN may affect the exposure of sensitive CYP3A4 substrates and/or narrow therapeutic index drugs that are initiated within 9 months after the last subcutaneous dose of LEN. # At least a 2-week (moderate inducers) or 4-week (strong inducers) cessation period is recommended prior to initiation of LEN due to the persisting inducing effect after discontinuation of an inducer. **Further Information.** For additional drug-drug interactions and for more detailed pharmacokinetic interaction data and dosage adjustments, please refer to:*http://www.hiv-druginteractions.org*(University of Liverpool)*.

For treatment, the 2022 PH ESC/ERS guidelines^1^ recommend initiation of ART according to current HIV guidelines as well as PAH-specific monotherapy, followed by sequential combination agents. HIV-PAH treatment follows idiopathic PAH principles, but data is largely limited to observational and open-label studies. Calcium channel blocker therapy should be avoided since HIV-PAH is not associated with favorable vasoreactive responses. Drug interactions, especially with protease inhibitor- or cobicistat-based ART and new long-acting injectables like lenacapavir, are key considerations in managing PAH-specific therapies^43^ (Figure 3). With ART and PAH-targeted treatment, outcomes of HIV-PAH now approximate those of idiopathic PAH.

## High-altitude PH and PAH

**Figure 4:**
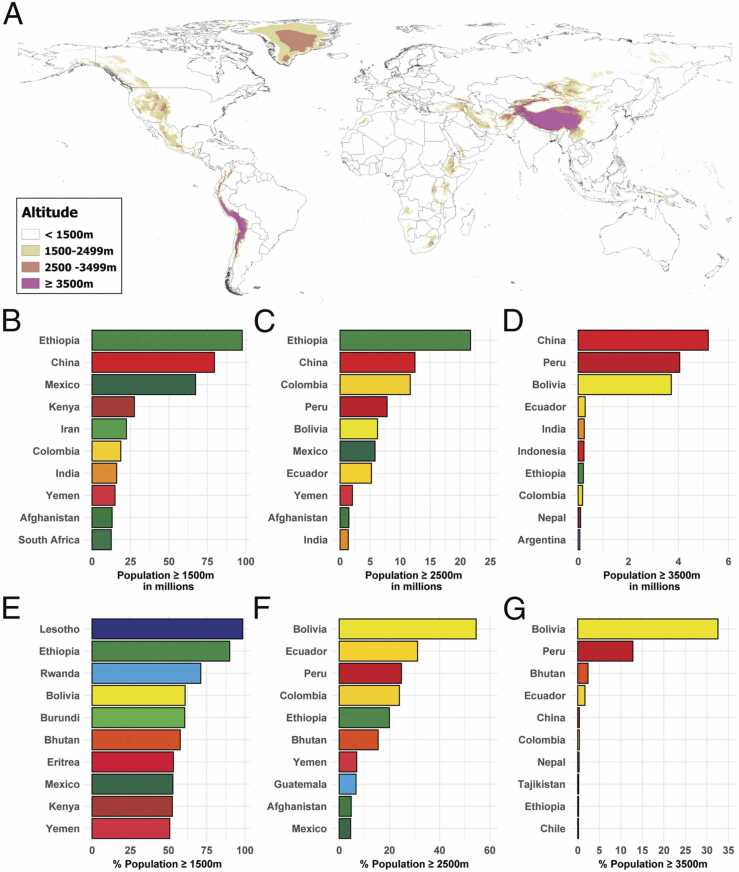
Global view of population living at high altitude 2021. Reproduced with permission by Dr. Tremblay JC (“Global and country-level estimates of human population at high altitude”. Proc Natl Acad Sci USA 2021;118.).

Over 81 million people live at high altitudes (≥2500 m), with the highest population density in Latin America^44^ (Figure 4). Hypobaric hypoxia induces vasoconstriction, which, when sustained, may lead to pulmonary vascular remodeling and PH. At high altitudes, this can manifest as either **high-altitude PH** (HAPH) or **high-altitude PAH** (HA-PAH), two distinct clinical entities.^45^

**HAPH** is defined by a mean pulmonary arterial pressure (mPAP) >30 mmHg on right heart catheterization or a systolic PAP >60 mmHg on echocardiography, thresholds that exceed the current PH definition of mPAP >20 mmHg.^46^ An evidence-based consensus definition of PH at high altitudes is still needed. In the South American Altiplano, HAPH affects an estimated 5–18% of residents living above 3200 m. In China, it is more prevalent in children than adults.^47^ The pathophysiology involves chronic hypoxia and dysregulation of the hypoxia-inducible factor (HIF) pathway. Under normoxia, HIFs are hydroxylated and degraded, whereas hypoxia stabilizes HIF-α subunits, triggering transcriptional activity that increases endothelin and angiotensin II levels while reducing nitric oxide (NO).^48^

HAPH can develop in individuals who relocate from low to high altitudes. Early stages may be asymptomatic, but as it progresses, symptoms such as dyspnea, fatigue, chest pain, syncope, and exercise intolerance typically emerge.^47^ Chest x-ray, electrocardiogram, and physical exam often reveal findings consistent with PH, while echocardiography serves as a useful screening tool. If treatment is considered, right heart catheterization is recommended for confirmatory diagnosis. Management involves relocation to lower altitude and supportive care for right heart failure. Few randomized controlled trials have evaluated PAH therapies — such as sildenafil, bosentan, fasudil, and nifedipine — in this context.^49^ Thus, more randomized controlled trials are needed to evaluate the effectiveness of PAH specific therapies in HAPH.

**HA-PAH** is a rare form of PAH occurring in individuals living at high elevations, typically above 2500 m. Unlike HAPH, HA-PAH is characterized by severe, progressive pulmonary vascular remodeling that persists despite descent to lower altitudes, often requiring long-term pharmacologic therapy. Epidemiological and treatment data for HA-PAH are largely derived from studies conducted at low altitudes. Preliminary estimates suggest that HA-PAH may affect millions of individuals living in high-altitude regions.^50^ Recently, two studies have described the characteristics of HA-PAH. Hoyos *et al*^51^ evaluated the demographic and clinical data from 36 patients living in Ecuador at an altitude of 2831 ±58 m with either PAH or CTEPH. Compared to European patients, their cohort was younger (mean age 44 ±13 years) with stable risk profiles despite severe hemodynamic impairment. Though limited by drug availability, treatment responses were favorable.^51^ Conde-Camacho *et a*l^52^ reported on a cohort of 188 patients from Colombia who resided permanently at altitudes >2500 m; 67% received specific PAH-therapy. Hemodynamic profile was similar to that seen in other PAH groups, with a 5 year-survival of 86.8%.

In summary, while HAPH is often caused by hypoxic pulmonary vasoconstriction and is generally reversible with descent to lower altitudes, HA-PAH involves fixed pulmonary vascular remodeling, sharing pathologic features with idiopathic PAH. HA-PAH persists despite removal from high altitude, often requiring long-term PAH-targeted therapy. Future comparative studies across altitudes and populations could clarify the role of hypoxia in PH and PAH, and improve treatment strategies.

## Conclusion

While idiopathic and common associated forms of PAH dominate clinical practice, rare causes represent a diverse and diagnostically challenging group. A thorough understanding and systematic evaluation of these conditions can facilitate early diagnosis, individualized treatment, and improved outcomes.

## Disclosures

None.

## Declaration of Competing Interest

The authors declare that they have no known competing financial interests or personal relationships that could have appeared to influence the work reported in this paper.
